# Autonomic and Neural Readiness for Social Threats in Patients with Social Anxiety Disorder

**DOI:** 10.1192/j.eurpsy.2025.377

**Published:** 2025-08-26

**Authors:** S.-Y. Kim, S. W. Kim, D. Lee, J. H. Kim, J. Lee, D. H. Kang, S.-H. Choi

**Affiliations:** 1 Psychology, Duksung Women’s University; 2 Seoul National University Hospital, Seoul, Korea, Republic Of

## Abstract

**Introduction:**

Pathological anxiety in social anxiety disorder (SAD) is characterized by dysregulated arousal and altered cardiac autonomic responses, with lower heart rate variability (HRV) potentially indicating emotional dysregulation.

**Objectives:**

This study aimed to explore the relationship between peripheral and central autonomic nervous system activity during emotional processing in patients with SAD.

**Methods:**

Thirty-two patients with SAD and 41 healthy controls participated in a passive viewing task that alternated between neutral and angry faces. We analyzed correlations between brain activation during emotional processing and root mean square of successive differences (RMSSD) in HRV during both resting state and task conditions.

**Results:**

Unlike the controls, the SAD group showed a trend toward significant correlations between baseline RMSSD and left anterior insula activity during neutral face processing (*R^2^* = 0.118, *β* = -0.003, *F= 3.886*, *p* = .058) and significant correlations with both left anterior insula and right amygdala activities during angry face processing (*R^2^* = 0.157, *β* = -0.003, *F*= 5.415, *p* = .027 and *R^2^* = 0.135, *β* = -0.002, *F*= 4.360, *p* = .046, respectively). In the control group, task RMSSD was significantly correlated with right amygdala and right dorsomedial prefrontal cortex activities during neutral face processing (*R^2^* = 0.160, *β* = -0.003, *F*=6.284, *p* = .017 and *R^2^* = 0.222, *β* = -0.009, *F*=9.443, *p* = .004, respectively), while in the SAD group, correlations were found with the right parahippocampal gyrus (*R^2^* = 0.148, *β* = -0.002, *F*=4.5, *p* = .044). Additionally, only in the control group, RMSSD during neutral face trials was significantly correlated with neural activation during angry faces processing (*R²* = 0.132, *β* = -0.002, *F*=4.856, *p* = .035).

**Conclusions:**

This study identifies distinct patterns of autonomic and neural responses to emotional stimuli in SAD patients, highlighting heightened autonomic readiness and reduced flexibility when processing social threats.

**Disclosure of Interest:**

None Declared

